# Content validity and psychometric properties of the inFLUenza Patient-Reported Outcome Plus (FLU-PRO Plus^©^) instrument in patients with COVID-19

**DOI:** 10.1007/s11136-022-03336-3

**Published:** 2023-01-27

**Authors:** Tom J. H. Keeley, Sacha Satram, Parima Ghafoori, Carolina Reyes, Helen J. Birch, Kimberly Raymond, Heather L. Gelhorn, Mark Kosinski, Cory D. Saucier, April Mitchell Foster, Amanda Lopuski, John H. Powers

**Affiliations:** 1 GlaxoSmithKline, 980 Great West Road, Brentford, Middlesex TW8 9GS UK; 2 Vir Biotechnology, San Francisco, CA USA; 3 GlaxoSmithKline, Collegeville, PA USA; 4 QualityMetric Incorporated, LLC, Johnston, RI USA; 5 Evidera, Bethesda, MD USA; 6 George Washington University School of Medicine, Washington, DC USA

**Keywords:** COVID-19, FLU-PRO Plus, Patient-reported outcome, Psychometric, Validity, Reliability

## Abstract

**Purpose:**

A well-defined and reliable patient-reported outcome instrument for COVID-19 is important for assessing symptom severity and supporting research studies. The InFLUenza Patient-Reported Outcome (FLU-PRO) instrument has been expanded to include loss of taste and smell in the FLU-PRO Plus, to comprehensively cover COVID-19 symptoms. Our studies were designed to evaluate and validate the FLU-PRO Plus among patients with COVID-19.

**Methods:**

Two studies were conducted: (1) a qualitative, non-interventional, cross-sectional study of patients with COVID-19 involving hybrid concept elicitation and cognitive debriefing interviews; (2) a psychometric evaluation of the measurement properties of FLU-PRO Plus, using data from COMET-ICE (COVID-19 Monoclonal antibody Efficacy Trial—Intent to Care Early).

**Results:**

In the qualitative interviews (*n* = 30), all 34 items of the FLU-PRO Plus were considered relevant to COVID-19, and participants determined the questionnaire was easily understood, well written, and comprehensive. In the psychometric evaluation (*n* = 845), the internal consistency reliability of FLU-PRO Plus total score was 0.94, ranging from 0.71 to 0.90 for domain scores. Reproducibility (Day 20–21) was 0.83 for total score, with domain scores of 0.67–0.89. Confirmatory factor analysis with the novel smell/taste domain demonstrated an acceptable fit to the data.

**Conclusion:**

The content, reliability, validity, and responsiveness of the FLU-PRO Plus in the COVID-19 population were supported. Our results suggest that FLU-PRO Plus is a content- and psychometrically-valid, fit-for-purpose measure which is easily understood by patients. FLU-PRO Plus is a suitable PRO measure for evaluating symptoms of COVID-19 and treatment benefit directly from the patient perspective.

**Trial Registration:** ClinicalTrials.Gov: NCT04545060, September 10, 2020; retrospectively registered.

**Supplementary Information:**

The online version contains supplementary material available at 10.1007/s11136-022-03336-3.

## Plain english summary

To assess how COVID-19 affects the lives of patients, researchers need to develop standard ways to measure its impact. An example of one of these measures is the FLU-PRO questionnaire, which was developed to assess the intensity and duration of symptoms in viral respiratory tract illnesses, such as influenza. Questions about loss of smell and taste, which are common symptoms of COVID-19, have been added to the FLU-PRO questionnaire, in an updated version named FLU-PRO Plus. In this study, we performed interviews to explore the symptom burden of COVID-19 and evaluate how relevant, important, and easily understood all the questions included in the FLU-PRO Plus are to patients who have recently tested positive for COVID-19. We also performed statistical analyses to determine the reliability and validity of the questionnaire. Our results show the COVID-19 symptoms measured by the FLU-PRO Plus are important and relevant to patients, and questions were easy to comprehend and covered all their symptoms, allowing an accurate depiction of the disease’s symptoms. We also found the FLU-PRO Plus was reliable, valid, and responsive to change. Findings from this study support the use of the FLU-PRO Plus in clinical trials and other research to assess COVID-19 symptoms and the impact of treatments on the disease, directly from the patient perspective.

## Introduction

A significant health burden is associated with coronavirus disease 2019 (COVID-19) [3]. To fully capture the symptom burden of COVID-19 on patients, comprehensive, standardized, and valid patient-reported outcome (PRO) instruments that reliably assess symptom severity are essential. In addition, well-defined and reliable PRO measures support therapy and vaccine effectiveness studies, alongside other research activities [4].

Epidemiological studies show that viral respiratory diseases have common symptom profiles, including cough, shortness of breath, fatigue, sore throat, muscle pain or body aches, headache, vomiting, and diarrhea [5]. The original inFLUenza Patient-Reported Outcome (FLU-PRO) measure was developed to assess core symptoms of influenza and other viral respiratory diseases, based on a recall period of the past 24 h [6, 7]. FLU-PRO consists of 32 items across six body systems (nose, throat, eyes, chest/respiratory, gastrointestinal, and body/systemic) which are predominantly scored on a five-point severity scale [6], and has been validated among patients with influenza and influenza-like illness [7, 8]. An extended version, the FLU-PRO Plus, has since been developed, which includes the commonly reported COVID-19 symptoms of loss of taste and smell [9]. FLU-PRO Plus has recently been shown to have good construct validity, known-groups validity, and responsiveness to change among patients with COVID-19 [4].

At initiation of this work, the psychometric properties of the FLU-PRO Plus had not yet been evaluated among the COVID-19 population. Two studies were therefore conducted to assess the measurement properties of FLU-PRO Plus among patients with COVID-19. First, a qualitative, cross-sectional, descriptive, non-interventional study was conducted, using an adapted grounded theory approach, to explore and gain insight into how patients describe their experience of COVID-19, and to identify COVID-19 symptoms that are important and clinically relevant to patients. In addition, the study aimed to elicit patient feedback on the relevance, comprehensiveness, and understandability of the FLU-PRO Plus when measuring COVID-19 symptoms. The second was a quantitative study that evaluated the factor structure and psychometric properties (reliability, construct and known-groups validity, responsiveness, and responder definition) of the FLU-PRO Plus for use in patients with COVID-19 in the COVID-19 Monoclonal antibody Efficacy Trial—Intent to Care Early (COMET-ICE) study population. COMET-ICE was a randomized, double-blind, multi-center, placebo-controlled trial that investigated the efficacy and safety of sotrovimab for the prevention of progression of mild/moderate COVID-19 in a high-risk adult population (NCT04545060).

## Methods

### Qualitative study

Hybrid concept elicitation and cognitive debriefing interviews were conducted among symptomatic adults with a confirmed diagnosis of COVID-19. Patients with mild, moderate, and severe COVID-19 were included in the sample. One-to-one, 90-min interviews were conducted via webcam or telephone. In the concept elicitation segment, participants described their experience of COVID-19, including its symptoms and impacts. In the cognitive debriefing segment, participants completed the FLU-PRO Plus questionnaire using a retrospective think-aloud method. A full description of the sample, procedure, and analysis of qualitative data is included in Online Resource 1.

### Quantitative analysis

Blinded PRO data from the COMET-ICE randomized clinical trial were used for the psychometric analysis, which was conducted over 12 weeks, using a data cut taken when all patients had reached Day 29 (full details of the COMET-ICE trial protocol are published elsewhere [10, 11]). Using an electronic device or paper questionnaire, participants completed the FLU-PRO Plus daily during the first 3 weeks following trial enrollment, and then on a single day at Weeks 4, 8, and 12. Participants also completed the 12-item Short Form (SF-12) hybrid survey [12] (the 12 items of the SF-12 plus the full Vitality and Role Physical Domains of the 36-item Short Form survey), which consists of eight domains and two summary scores. An additional pre-COVID health supplemental question (“Compared to before the COVID-19 crisis, how would you rate your health in general now?”) was added for the purpose of this study. Scores are transformed to a metric, with scores > 50 indicating better physical or mental health than the mean. The Work Productivity and Activity Impairment Questionnaire: General Health (WPAI-GH)﻿ [13] was also completed, which assesses absenteeism, presenteeism, work productivity loss, and activity impairment across six items. WPAI-GH scores are expressed as impairment percentages, with higher numbers indicating greater impairment and less productivity. Both questionnaires were distributed at key timepoints (Weeks 1 [Day 1], 2 [Day 15], 4 [Day 29], 8, 12, 16, 20, and 24).

### Psychometric evaluation

The psychometric evaluation of the FLU-PRO Plus was performed in accordance with classical test theory [14] and comprised two phases. Phase I involved confirmation of factor structure, including item evaluation and scaling, and evaluation of the instrument’s fit with the inclusion of additional COVID-19-specific items (i.e., loss of taste and loss of smell). Phase II involved assessment of the reliability, validity, and responsiveness of the measure.

Descriptive statistics (*N*, mean, standard deviation [SD]) were calculated for FLU-PRO Plus items across all 21 days in which the FLU-PRO Plus was administered, and for the SF-12 and WPAI-GH. Both FLU-PRO and FLU-PRO Plus scores are reported for the analyses below, in order to assess consistency between the measures.

Confirmatory factor analysis (CFA) models were run to evaluate the fit of the original FLU-PRO and FLU-PRO Plus single-factor and multi-factor conceptual models for use in the COVID-19 population (factor structure). CFA was conducted using Mplus software [15]; CFA model fit was assessed with the *χ*^2^ test, comparative fit index (CFI), root mean squared error of approximation (RMSEA), and standardized root mean square residual (SRMR). A low *χ*^2^ value relative to the degrees of freedom indicates a better fit [16]. Acceptable model fit is indicated when CFI > 0.90, and RMSEA or SRMR < 0.07 [17, 18].

Reliability of the FLU-PRO Plus was assessed for internal consistency and reproducibility. Internal consistency was evaluated using Cronbach’s coefficient alpha at Day 1, with descriptive scores from 0 to 1.0 and higher scores indicating a more homogenous instrument [14, 19]. The reproducibility of FLU-PRO domain and total scores was evaluated *post hoc* among participants with no change in hospitalization status between Days 20 and 21, and who had FLU-PRO Plus data on both days. Reproducibility was assessed using an estimation of an intraclass correlation coefficient (ICC) and a calculation of effect size from Days 20 to 21 with a two-way mixed-effect analysis of variance model. ICC values range from 0 to 1, with ICC > 0.6 generally considered acceptable [20, 21].

Construct validity assessed the relationship between FLU-PRO Plus and other PRO measures (SF-12 [mental and physical component summary scores, role physical, and vitality domains and general health question], and WPAI-GH [percent impairment, work productivity, and regular activities]). Construct validity of the FLU-PRO Plus was assessed at Days 1, 15, and 29 using Spearman correlations. A correlation coefficient > 0.3 indicates convergent validity [22].

Known-groups validity and responsiveness were assessed through an analysis of variance and an analysis of covariance, respectively, using WPAI activity impairment score as a variable. Responsiveness was assessed by comparing changes from Day 1 to Days 7, 14, and 21 in WPAI-GH scores, using an analysis of covariance.

A pre-specified responder definition for the FLU-PRO Plus, developed through discussion with regulatory authorities, comprised key COVID-19 symptoms as understood at the time. This definition allowed for persistent cough and fatigue (responses up to “Somewhat”) and loss of taste and smell to continue but required other symptoms to resolve (full details of the responder definition in Supplementary Table 1 [Online Resource 1]). To explore the value of the responder definition, comparisons between FLU-PRO Plus responders and non-responders were made using the SF-12 scales, pre-COVID health supplemental question, and WPAI scores at Days 15 and 29, and Weeks 8 and 12.

### Statistical methods

All statistical tests used a significance level of 0.05 unless otherwise stated. Statistical tests involving multiple comparisons were adjusted for multiplicity to reduce the possibility of Type I error. For continuous variables, mean, median, SD, and range are described. For categorical variables, frequency and percentage are described.

PRO data missing due to early withdrawal from the study, a missed entry, device failure, or non-compliance were not included in the psychometric analyses. No item-level missing data were expected for data collected with electronic devices, as participants were required to select a response before advancing to subsequent items. Due to the rapid initiation of sites, paper completion of questionnaires was sometimes required. Paper records with missing data were scored according to the original FLU-PRO user manual. To score the novel “Smell/taste” domain of FLU-PRO Plus, data on at least one of the two items were required.

## Results

### Qualitative analysis

#### Study participants

A total of 30 symptomatic patients with a confirmed COVID-19 diagnosis participated in the interviews, which were conducted an average of 34.9 (SD = 15.0; range: 12–66) days after testing. Participants were evenly split in terms of sex and severity of symptoms (mild or moderate/severe) (Table 1). Mean age (range) was 49.8 (22–70) years and most participants (70%) were White. Of the 30 patients included in the sample, 50.0% had mild COVID-19, 33.3% had moderate COVID-19, and 16.7% had severe COVID-19, with 36.7% of participants also having comorbid conditions including diabetes, asthma, and Crohn’s disease. The saturation analysis did not identify any new symptoms in the last code sets, suggesting sufficient interviews were conducted to reach saturation.

**Table 1:** Study participants (qualitative analysis)

Variable	Overall (*N* = 30)
Mean age (range), years	49.8 (22–70)
Age group, *n* (%)	
18–27	1 (3.3)
28–37	6 (20.0)
38–54	8 (26.6)
55–57	7 (23.3)
58–67	5 (16.7)
68–77	3 (10.0)
Sex, *n* (%)	
Male	15 (50.0)
Female	15 (50.0)
Race/ethnicity, *n* (%)	
Asian	2 (6.7)
Black	3 (10.0)
Hispanic or Latino	4 (13.3)
White	21 (70.0)
Geographic region	
Northeast/Mid-Atlantic (MA, NY, NJ, PA)	9 (30.0)
Southeast (FL, GA, NC, TN)	7 (23.3)
South (TX)	5 (16.7)
Midwest (IL, MN, MO)	4 (13.3)
West (AZ, CA, CO)	5 (16.7)
Education	
High school diploma or GED	2 (6.7)
Some college	4 (13.3)
AD, BA, or technical certificate	16 (53.3)
Graduate degree (MA, MS, PhD, MD)	8 (26.7)
Comorbid conditions, *n* (%)	
Yes	11 (36.7)
No	19 (63.3)
COVID-19 severity status	
Mild	15 (50.0)
Moderate	10 (33.3)
Severe	5 (16.7)
Mean number of symptoms (range)	7.3 (3–12)
Time from diagnosis to interview, days	
Mild	29.4
Moderate	40.2
Severe	40.6

*AD* associate degree, *AZ* Arizona, *BA* Bachelor of Arts, *CA* California, *CO* Colorado, *COVID-19* coronavirus disease 2019, *FL* Florida, *GA* Georgia, *GED* General Educational Development, *IL* Illinois, *MA﻿* Maine, *MA* Master of Arts, *MD* Doctor of Medicine, *MN* Minnesota, *MO* Missouri, *MS* Master of Science, *NC* North Carolina, *NJ* New Jersey, *NY* New York, *PA* Pennsylvania, *PhD* Doctor of Philosophy, *TN* Tennessee, *TX* Texas

#### COVID-19 symptoms and participant feedback on the FLU-PRO Plus

**Fig. 1:**
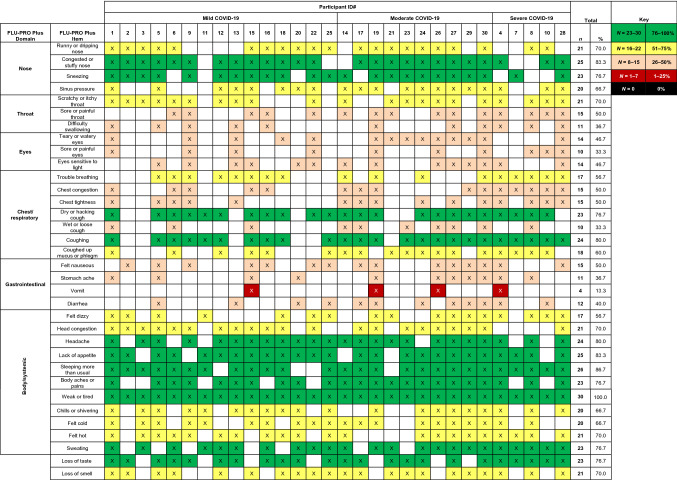
Frequency and proportion of FLU-PRO Plus symptoms reported by COVID-19 participants (qualitative analysis). “X” corresponds to a symptom elicited, either spontaneously or when probed, by a participant during the hybrid interview. Shading of cells corresponds to the proportion of the sample that elicited each symptom of the FLU-PRO Plus

**Fig. 2:**
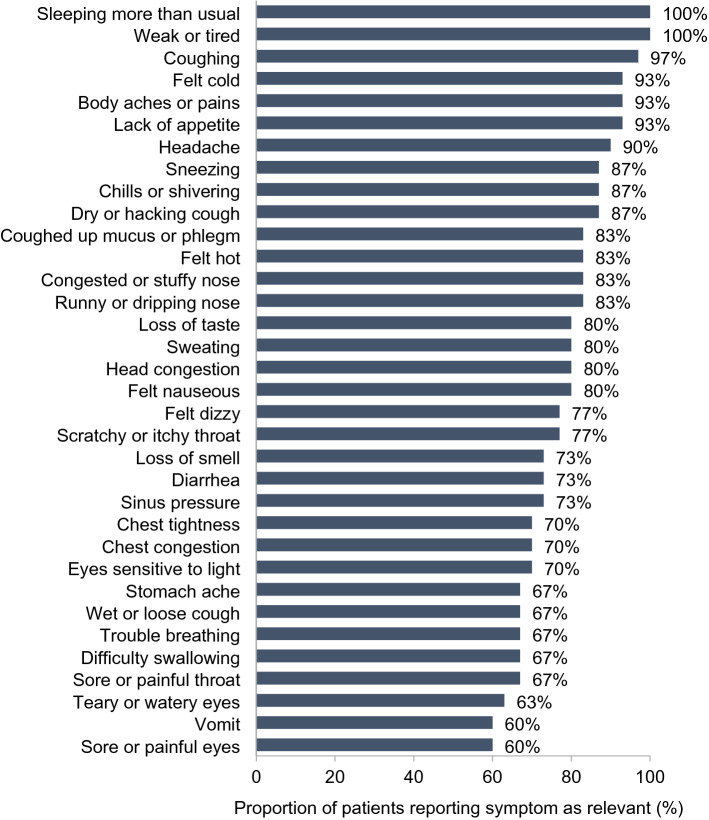
Endorsement levels of COVID-19 symptoms across interviews (qualitative analysis)

During qualitative interviews, participants described experiencing a wide array of COVID-19-related symptoms, either spontaneously or following probes, confirming the systemic and variable presentation of the disease. Participants described variability in the symptoms experienced, when symptoms occurred, and the duration of symptoms, highlighting the heterogenous nature of COVID-19. Figure 1 shows the proportion of participants who described experiencing each item of the FLU-PRO Plus questionnaire across different disease severities. Overall, most common symptoms were feeling weak or tired (100.0%), sleeping more than usual (86.7%), congested/stuffy nose (83.3%), and lack of appetite (83.3%). There was no clear pattern of symptom variation by severity, other than respiratory-related symptoms (which were reported by a higher percentage of severe participants). All severe participants (*n* = 5; 100.0%) reported trouble breathing and chest congestion, while the majority (*n* = 4; 80.0%) also reported symptoms of chest tightness and shortness of breath. Similarly, those with comorbid conditions reported a dry or hacking cough (*n* = 10; 90.9%), trouble breathing (*n* = 8; 72.7%), and shortness of breath (*n* = 7; 63.6%) more often than those without comorbid conditions. Due to the variability of symptoms experienced, each individual participant did not report that all symptoms in the FLU-PRO Plus were relevant to their personal experience. However, all 34 items received high levels of endorsement, with at least 60% of participants reporting each item as relevant to their experience of COVID-19 (Fig. 2). All items of the instrument mapped directly to the COVID-19 symptoms reported by participants (see Supplementary Table 2 [Online Resource 1] for exemplary quotes).

Overall, participants found the FLU-PRO Plus to be well written and comprehensible (Table 2). Only one item pertaining to the symptom “head congestion” was reported as difficult to comprehend by four participants (13.3%), since the item could be misinterpreted to be a symptom similar to “brain fog” by those participants.

**Table 2:** Study participants’ feedback on the FLU-PRO Plus questionnaire (qualitative analysis)

FLU-PRO Plus aspect	Endorsed, *n* (%) *N* = 30
Instructions were simple and easy to understand	27 (90)
Length of questionnaire was appropriate	26 (86)
Response options were clear, understandable, and appropriate	23 (76)
Questionnaire was comprehensive	22 (73)
Recall period of 24 h was appropriate	20 (66)

*FLU-PRO* InFLUenza Patient-Reported Outcome

All 34 items were considered relevant and important to capture the heterogeneity of COVID-19 symptoms. While most participants (73.3%) indicated that the FLU-PRO Plus comprehensively captured their experience, 14 participants (46.7%) mentioned experiencing a disturbance in their thinking and cognitive capacity while ill, which was termed “brain fog” by some. This impact of COVID-19 was not covered by the FLU-PRO Plus.

Participants agreed the FLU-PRO Plus instructions were simple and easy to understand (*n* = 27; 90%), and most reported that the length of the questionnaire was appropriate for capturing COVID-19 symptoms (*n* = 26; 86%). There was general agreement that the 24-h recall period would be easy and useful to report COVID-19 symptoms. The majority of participants (*n* = 23; 76%) found the response options (Not at all, A little bit, Somewhat, Quite a bit, Very much) which were used for most FLU-PRO Plus items to be appropriate. A Yes/No response option to loss of smell and taste items was also reported as adequate by 90% of participants; one participant suggested a scale could be more useful if the response options reflected a partial loss, decrease, or change in these items.

### Quantitative analysis

#### Patients

Of the 1057 patients enrolled in COMET-ICE at the time of the analyses, 845 had FLU-PRO Plus score data at Day 1 and at least one follow-up visit, and were therefore included in the psychometric analyses. Mean (SD) age was 52.3 (14.9) years, 55.3% of patients were female, and the majority were White (87.1%) (Table 3). Of the 845 patients, the proportion included in the analyses who completed the FLU-PRO Plus questionnaire was 75.0% at Day 2, 53.7% at Day 21, 62.8% at Day 29, 58.2% at Week 8, and 48.2% at Week 12.

**Table 3:** Study participants (quantitative analysis)

Variable	Overall (*N* = 845)
Mean (SD) age [range], years	52.3 (14.9) [78]
Sex, *n* (%)	
Male	378 (44.7)
Female	467 (55.3)
Ethnicity, *n* (%)	
Hispanic or Latino	551 (65.2)
Not Hispanic or Latino	294 (34.8)
Race, *n* (%)	
American Indian or Alaska Native	2 (0.2)
Asian	33 (3.9)
Black or African American	70 (8.3)
Native Hawaiian or Other Pacific Islander	0
White	736 (87.1)
Multiple races	2 (0.2)
Missing	2 (0.2)
Medical conditions, *n* (%)	
Diabetes	177 (20.9)
Congestive heart failure	6 (0.7)
Chronic kidney disease	10 (1.2)
Moderate/severe asthma	137 (16.2)
Chronic obstructive pulmonary disease	53 (6.3)

*SD* standard deviation

#### FLU-PRO Plus item evaluation and scaling

**Fig. 3:**
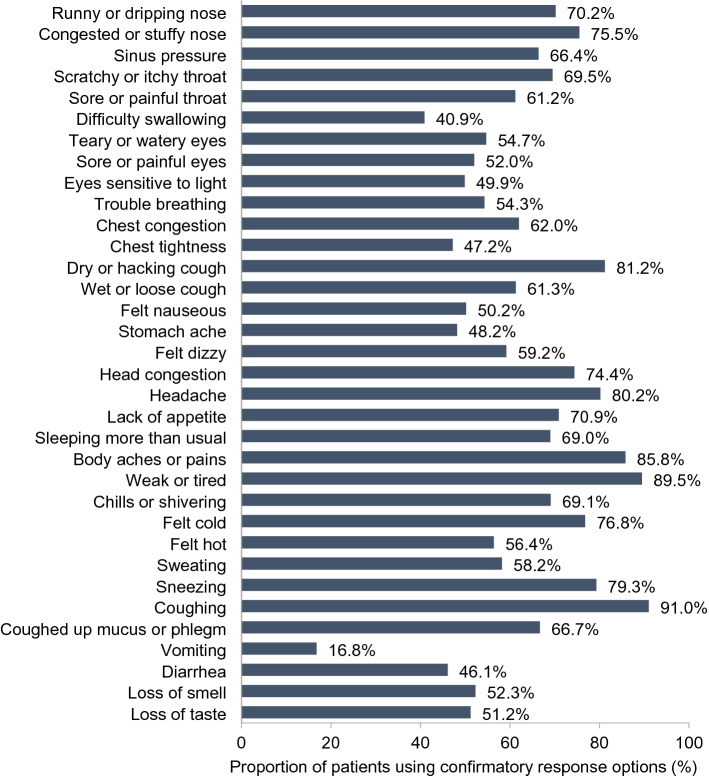
FLU-PRO Plus item confirmatory response frequency at Day 1 (psychometric analysis). Frequency of item responses “A Little Bit”, “Somewhat”, “Quite a Bit”, and “Very Much” were summed to give the total frequency of confirmatory option selection. Response options for Vomiting and Diarrhea of  ≥ 1 incident were classed as confirmatory. Response options for Loss of smell and Loss of taste of “Yes” were classed as confirmatory

**Fig. 4:**
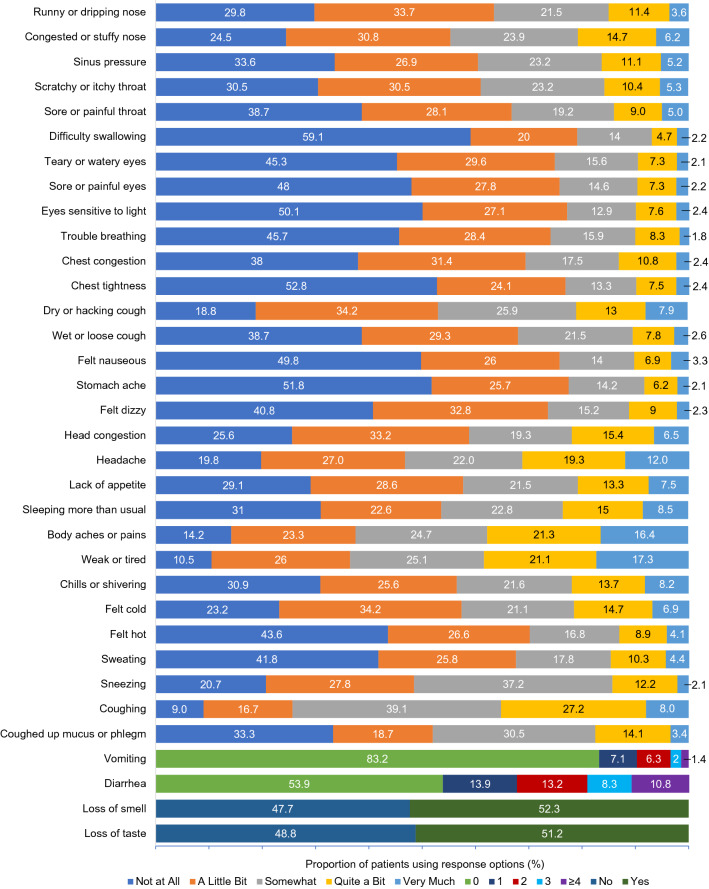
FLU-PRO Plus item response frequency distribution at Day 1 (psychometric analysis). Different response options were given for some items of the FLU-PRO Plus. A scale from “Not at All” to “Very Much” was used for most items in Fig. 4. Where values of 0, 1, 2, 3, and ≥ 4 were used, these indicate the number of times the patient experienced vomiting or diarrhea. Loss of smell and taste were measured by either “Yes” or “No” response options

For the quantitative analysis, mean FLU-PRO Plus item scores ranged from 0.3 (vomiting) to 2.1 (weak or tired and coughing) at Day 1, and most items were experienced by more than 50% of patients (Fig. 3). There was good use of the range of response options, and at least some participants endorsed each of the response options for every symptom (Fig. 4). In addition, there were some expected floor effects due to the heterogeneity of COVID-19 symptoms. Mean item scores reduced over time but a range of response options continued to be selected and floor effects were maintained, with less severe response options selected more frequently at later trial timepoints.

At ﻿Day 1, WPAI-GH scores were high and SF-12 scores were low, indicating a substantial impact of COVID-19 on patients, but were followed by notable improvements by Day 15 and through Week 12.

#### Confirmatory analysis

The multi-factor models (defined based on the conceptual models for the original FLU-PRO [six factors] and FLU-PRO Plus [seven factors]) yielded an acceptable fit to the data, with factor loadings > 0.4 for all items, and > 0.7 for most items (Table 4).

**Table 4:** Confirmatory factor analyses for FLU-PRO and FLU-PRO Plus (Day 3) (quantitative analysis)

Domain	Item number	Original FLU-PRO—single factor	Original FLU-PRO plus two COVID items (32 + 2)— single factor	Original FLU-PRO with domain structure			Original FLU-PRO with domain structure and placement of COVID items	
Nose	1. Runny or dripping nose	0.637	0.633	0.833	0.753		0.832	0.753
	2. Congested or stuffy nose	0.719	0.715		0.829			0.830
	3. Sinus pressure	0.777	0.772		0.916			0.915
	28. Sneezing	0.586	0.582		0.710			0.709
Throat	4. Scratchy or itchy throat	0.783	0.778	0.784	0.899		0.782	0.897
	5. Sore or painful throat	0.837	0.833		0.947			0.947
	6. Difficulty swallowing	0.785	0.783		0.924			0.926
Eyes	7. Teary or watery eyes	0.717	0.713	0.826	0.863		0.826	0.862
	8. Sore or painful eyes	0.756	0.753		0.891			0.891
	9. Eyes sensitive to light	0.718	0.716		0.846			0.847
Chest/respiratory	10. Trouble breathing	0.751	0.747	0.775	0.854		0.773	0.856
	11. Chest congestion	0.811	0.806		0.900			0.899
	12. Chest tightness	0.795	0.789		0.882			0.881
	13. Dry or hacking cough	0.649	0.645		0.750			0.751
	14. Wet or loose cough	0.707	0.703		0.804			0.805
	29. Coughing	0.726	0.719		0.837			0.836
	30. Coughed up mucus or phlegm	0.682	0.675		0.776			0.776
Gastrointestinal	15. Felt nauseous	0.713	0.712	0.874	0.843		0.876	0.844
	16. Stomach ache	0.732	0.729		0.869			0.867
	31. How many times did you vomit?	0.645	0.649		0.763			0.767
	32. How many times did you have diarrhea?	0.392	0.386		0.467			0.463
Body/systemic	17. Felt Dizzy	0.697	0.698	0.876	0.755		0.878	0.758
	18. Head congestion	0.744	0.741		0.806			0.805
	19. Headache	0.715	0.711		0.768			0.768
	20. Lack of appetite	0.652	0.651		0.700			0.703
	21. Sleeping more than usual	0.582	0.578		0.629			0.628
	22. Body aches or pains	0.761	0.759		0.815			0.816
	23. Weak or tired	0.725	0.721		0.775			0.774
	24. Chills or shivering	0.883	0.880		0.919			0.919
	25. Felt cold	0.852	0.848		0.883			0.881
	26. Felt hot	0.715	0.715		0.769			0.771
	27. Sweating	0.685	0.683		0.738			0.739
Smell/taste	33. Loss of smell	–	0.664	–	–		0.482	0.967
	34. Loss of taste	–	0.692	–	–			0.967
Model fit statistics								
*χ*^2^ (df)		4911.492 (464)	6180.592 (527)	2171.070 (458)		2204.428 (520)		
*P*-value		< 0.0001	< 0.0001	< 0.0001		< 0.0001		
CFI		0.794	0.753	0.921		0.927		
RMSEA (90% CI)		0.128 (0.125–0.131)	0.135 (0.132–0.138)	0.080 (0.077–0.083)		0.074 (0.071–0.077)		
SRMR		0.087	0.100	0.059		0.059		

A low *χ*^2^ value relative to the degrees of freedom indicates better fit [16]. Acceptable model fit is indicated when CFI > 0.90, and RMSEA or SRMR < 0.07 [17, 18]

*CFI* comparative fit index, *CI* confidence interval, *COVID-19* coronavirus disease 2019, *df* degrees of freedom, *FLU-PRO* InFLUenza Patient-Reported Outcome, *RMSEA* root mean square error of approximation, *SRMR* standardized root mean residual, *χ*^*2*^ chi-square test

#### FLU-PRO Plus assessment of reliability, validity, and responsiveness

At Day 1, internal consistency reliability (Cronbach’s coefficient alpha) for the original FLU-PRO and FLU-PRO Plus total scores was 0.95 and 0.94, respectively. Cronbach’s coefficient alpha for the FLU-PRO Plus domain scores ranged from 0.71 (gastrointestinal) to 0.90 (body/systemic); the smell/taste domain score was 0.86.

In the *post hoc* reproducibility analysis conducted using data from Day 20 to 21, ICCs were good for both total scores and all domains. For total scores, ICCs were 0.82 and 0.83 for FLU-PRO and FLU-PRO Plus, respectively. For domain scores, ICCs were 0.89 for smell/taste, 0.84 for throat, 0.82 for nose and chest/respiratory, 0.81 for body/systemic, 0.68 for eyes, and 0.67 for gastrointestinal.

An analysis of construct validity at Day 1 is presented in Table 5. Moderate correlations between FLU-PRO Plus total score and SF-12 (mental and physical components, and role physical domain) scores were observed at Days 1, 7, and 15 (r range: − 0.37 to − 0.55). Moderate correlations were observed between FLU-PRO Plus total score and the WPAI-GH (r range: 0.41 to 0.58).

**Table 5:** Construct validity: FLU-PRO Plus scale correlations with other PRO measures (Day 1) (quantitative analysis)

		Correlation coefficient								
PRO measure (Day 1)	*N*	Nose	Throat	Eyes	Chest/respiratory	Gastrointestinal	Body/systemic	Smell/taste	FLU-PRO total score	FLU-PRO Plus total score
SF-12 MCS	726	− 0.26^****^	− 0.25^****^	− 0.34^****^	− 0.28^****^	− 0.36^****^	− 0.40^****^	− 0.13^***^	− 0.40^****^	− 0.39^****^
SF-12 PCS	726	− 0.20^****^	− 0.24^****^	− 0.32^****^	− 0.37^****^	− 0.24^****^	− 0.43^****^	− 0.15^****^	− 0.42^****^	− 0.41^****^
SF-12 Role physical	726	− 0.23^****^	− 0.23^****^	− 0.31^****^	− 0.37^****^	− 0.30^****^	− 0.50^****^	− 0.17^****^	− 0.46^****^	− 0.45^****^
SF-12 Vitality	726	− 0.19^****^	− 0.07	− 0.08^*^	− 0.23^****^	− 0.12^**^	− 0.38^****^	− 0.01	− 0.29^****^	− 0.28^****^
SF-12 General health question	726	− 0.09^*^	− 0.15^****^	− 0.21^****^	− 0.22^****^	− 0.16^****^	− 0.18^****^	− 0.04	− 0.21^****^	− 0.21^****^
WPAI-GH Overall work impairment (%)	115	0.19^*^	0.25^**^	0.33^***^	0.35^***^	0.26^**^	0.40^****^	0.16	0.39^****^	0.39^****^
WPAI-GH Work time missed (%)	346	0.09	0.09	0.13^*^	0.16^**^	0.20^***^	0.15^**^	0.16^**^	0.18^***^	0.20^***^
WPAI-GH Activity impairment (%)	350	0.25^****^	0.31^****^	0.35^****^	0.32^****^	0.29^****^	0.45^****^	0.16^**^	0.44^****^	0.43^****^

Correlation coefficients were calculated using Spearman rank-order correlations. SF-12 scores are transformed into a z-score for comparison with normative data, with scores > 50 indicating better physical or mental health than the mean. WPAI-GH items are scored as percentages, with higher numbers indicating greater impairment and less productivity

*FLU-PRO* InFLUenza Patient-Reported Outcome, *MCS* mental component summary, *PCS* physical component summary, *PRO* patient-reported outcome, *SF-12* 12-item Short Form, *WPAI-GH* Work Productivity and Activity Impairment: General Health questionnaire

^*^p < 0.05; **p < 0.01; ***p < 0.001; ****p < 0.0001

Known-groups validity and responsiveness were also demonstrated. Analysis using the WPAI-GH—Activity Impairment showed significant known-groups validity for the original FLU-PRO and FLU-PRO Plus total scores (*p* < 0.0001) at all timepoints, and the domains showed similar results (Supplementary Tables 3–7 [Online Resource 1]). FLU-PRO Plus total score was responsive to change in WPAI-GH score from Day 1 to Day 29 (*n* = 173; *p* < 0.05 for all) (Supplementary Table 8 [Online Resource 1]). The a priori responder definition performed well. For all comparisons of all variables at each timepoint, responders had better scores than non-responders (*p* < 0.05), except for missed work time at Week 12 (Supplementary Tables 9–12 [Online Resource 1]).

## Discussion

Findings from this qualitative study and psychometric evaluation support the content validity and conceptual structure of the FLU-PRO Plus in the setting of COVID-19, indicating that the validity of the measure in COVID-19 is consistent with its demonstrated validity in other viral respiratory diseases. Furthermore, our study is among the first to explore COVID-19 symptoms and experience qualitatively, directly from the patient perspective, and we extend previous quantitative work to a greater number of participants with more severe disease.

Both the qualitative and quantitative studies tested and confirmed that FLU-PRO Plus is an appropriate and comprehensive tool for measuring COVID-19 symptoms and their improvement. We provide valuable qualitative evidence of high levels of participant endorsement of all FLU-PRO Plus items as relevant. Participants also confirmed they experienced symptoms in the manner described in the FLU-PRO Plus items and understood the meaning of each item appropriately.

The addition of two new items (loss of taste and loss of smell) to assess COVID-19 symptoms was supported by the data. These symptoms were endorsed during interviews and fit into the adapted conceptual framework of the FLU-PRO instrument as a separate domain (smell/taste), thus supporting their inclusion in the total FLU-PRO Plus score. The possibility of including an additional item to account for the “brain fog” noted by some participants may warrant further study, of both wording/response options and the investigation of “brain fog” as a multi-dimensional concept resulting from COVID-19 symptoms (rather than a symptom of COVID-19 itself).

Our analyses support the reliability, reproducibility, construct validity, known-groups validity, and responsiveness of the FLU-PRO Plus. FLU-PRO Plus scores declined throughout the trial as patients experienced improvement in their illness and were responsive to changes in WPAI-GH activity impairment score. This supports the overall validity of the FLU-PRO questionnaire to assess COVID-19 symptoms. This conclusion is supported by another recent study which showed FLU-PRO Plus was reliable, valid, and responsive to change in patients with COVID-19 [4].

Recently, FLU-PRO Plus was endorsed as an outcome measure by the International Consortium for Health Outcomes Measurement COVID-19 Working Group, further supporting its integration into research activities and in assessing COVID-19 symptoms [23]. Based on our findings and use of the FLU-PRO Plus in the COMET-ICE trial, the instrument is suitable for use in observational studies, clinical trials of COVID-19 treatments, and clinical practice with the purpose of evaluating COVID-19 symptoms and improvements, directly from the patient perspective. FLU-PRO Plus can also be used as an outcomes assessment in COVID-19 studies.

This work is consistent with good scientific principles and those articulated in the US Food and Drug Administration (FDA) Patient-Reported Outcome guidance [﻿^24^, ^25^]. The FDA has issued guidance for assessing COVID-19 in clinical trials, which includes 14 common symptoms [26]. FLU-PRO Plus consists of 34 items and encompasses all the common symptoms contained in the guidance. All items of the FLU-PRO Plus questionnaire received high levels of endorsement by participants during the qualitative interviews, and quantitative analyses demonstrated a good distribution across all item response categories. These data underscore the known symptom heterogeneity reported among patients with COVID-19 [27]. Furthermore, they highlight the importance of a comprehensive tool which covers the diversity of symptoms experienced, and the loss of information which will occur when using measures that focus solely on the clinician-identified “core symptoms” of COVID-19.

Other PRO instruments have since been designed to evaluate COVID-19 symptoms, such as the 23-item Symptoms Evolution of COVID-19 (SE-C19) [28]. The SE-C19 also uses a recall period of the past 24 h, and its response options include No symptoms, Mild, Moderate, and Severe. To validate the instrument, 30 non-hospitalized patients with COVID-19 participated in concept elicitation and cognitive debriefing interviews. Minor improvements to SE-C19 were suggested to improve conceptual clarity, including separating loss of smell/taste into two items, as in FLU-PRO Plus. The Symptom-Burden Questionnaire for Long COVID (SBQ™-LC) has also been recently developed to assess the symptom burden of “long” COVID-19 [29].

There are some potential limitations to the studies described here. In the qualitative analysis, all interviews had to be conducted remotely, instead of in-person, where nonverbal and behavioral nuances important for interpreting cognitive interviews can be detected. To mitigate this, interviewers were trained to listen for lengthy pauses, recognize changes in tone and inflection, and detect verbal indications of confusion, which could indicate challenges in understanding and/or responding to an item. Webcams were used whenever possible to facilitate face-to-face interaction. While technical familiarity prevented some participants from participating by webcam, no differences in responses across these two modes were noted. Due to the acute nature of COVID-19, most participants had largely recovered at interview, so symptoms were discussed retrospectively. However, all interviews were conducted within 66 days of a positive COVID-19 diagnosis, and participants did not experience difficulties remembering and discussing details of their illness. Additionally, purposive sampling methods were used to ensure that at least 20–30% of participants had pre-existing conditions that placed them in a higher risk category. While this was achieved, the primary comorbidities documented were obesity or diabetes, and so future studies should capture a greater range of pre-existing conditions. In addition, there were few participants aged ≥ 65 years, who are likely to be most severely impacted by COVID-19. Future analyses should investigate symptom burden among this group. In the quantitative analysis, use of a population with mild-to-moderate COVID-19 symptoms may limit the generalizability of the results to patients with more severe symptoms. Finally, due to the dynamic nature of the pandemic, COMET-ICE trial sites were initiated rapidly following aggressive timelines to study initiation. As a result, there was insufficient time to build electronic PRO instruments and so many patients completed questionnaires on paper before transitioning to electronic PRO measures, which affected response rate. However, only completed, available data were used in this psychometric evaluation, and therefore the impact of missing data on these findings is assumed to be minimal. In addition, these time constricts did not make inclusion of typical anchors used in a psychometric analysis such as this one possible, with the exception of the WPAI.

## Conclusion

The qualitative analysis supports the content validity of FLU-PRO Plus, in that the concepts measured are relevant and important to patients with COVID-19, and the questions and response options are understandable. The results of the psychometric analyses support the reliability, validity, and responsiveness of FLU-PRO Plus in individuals with symptoms of COVID-19. FLU-PRO Plus is a well-defined, reliable, and psychometrically sound measure with proven construct- and content-validity. Therefore, these findings indicate that FLU-PRO Plus is an appropriate tool for measuring symptoms of COVID-19 infection.

## Supplementary Information

Below is the link to the electronic supplementary material.

Supplementary file1 (DOCX 74 KB)

## Data Availability

Anonymized individual participant data and study documents can be requested for further research from https://www.clinicalstudydatarequest.com.
