# Supplementary material for: Content validity and psychometric properties of the inFLUenza Patient-Reported Outcome Plus (FLU-PRO Plus©) instrument in patients with COVID-19
Source: Qual Life Res. 2023 Jan 27;32(6):1645–57. doi: 10.1007/s11136-022-03336-3 (PMC9879742; doi:10.1007/s11136-022-03336-3)
Supplement: Supplementary file 1 — Supplementary file1 (DOCX 74 KB) [file 11136_2022_3336_MOESM1_ESM.docx]

# Online Resource 1

**Content validity and psychometric properties of the inFLUenza Patient-reported Outcome PLUS (FLU-PRO PLUS^©^) instrument in patients with COVID-19**

***Quality of Life Research***

**Authors:**

Tom J.H. Keeley^1^ ([0000-0003-1584-9093](https://orcid.org/0000-0003-1584-9093)), Sacha Satram^2^, Parima Ghafoori^3^, Carolina Reyes^2^, Helen J. Birch^1^ ([0000-0002-4924-4810](https://orcid.org/0000-0002-4924-4810)), Kimberly Raymond^4^, Heather L. Gelhorn^5^, Mark Kosinski^4^, Cory D. Saucier^4^ ([0000-0002-0420-6458](https://orcid.org/0000-0002-0420-6458)), April Mitchell Foster^4^, Amanda Lopuski^3^, John H. Powers III^6^

**Affiliations:**

^1^GlaxoSmithKline, Brentford, Middlesex, UK

^2^Vir Biotechnology, San Francisco, CA, USA

^3^GlaxoSmithKline, Collegeville, PA, USA

^4^QualityMetric Incorporated, LLC, Johnston, RI, USA

^5^Evidera, Bethesda, MD, USA

^6^George Washington University School of Medicine, Washington, DC, USA

**Corresponding author email:** tom.x.keeley@gsk.com

**Qualitative study methods**

### ***Sample and recruitment***

Participants were recruited in the United States (US) through an agency specializing in healthcare recruitment. The agency identified potential study participants from a pre-existing panel of patients, physician referrals, and broader social media outlets (e.g., Facebook). Individuals aged ≥18 years with a COVID-19 diagnosis confirmed by a positive laboratory polymerase chain reaction (PCR) or antigen test were eligible for inclusion. It was required that diagnoses be received at least 10 days prior to screening to ensure illness had progressed sufficiently to avoid misclassification of severity and allow the patient to describe their full disease experience, but within three months to guarantee accurate recall of symptoms. Participants also needed to experience symptomatic COVID-19 (with ≥2 symptoms), be able to read and speak English fluently, be willing to participate in a 90-minute interview by phone/webcam, and provide informed consent.

A sample size of 30 participants was determined based on commonly used standards regarding the number of interviews needed to test item understandability and concept relevance [1-3]. A purposive sampling approach was used to construct a sample whereby FLU-PRO Plus could be tested across individuals with varying severity levels of COVID-19, and to achieve variation in characteristics, such as geographic residence, sex, race, and age. Patients with mild, moderate, and severe disease were included in this sample. In this study, mild COVID-19 was defined as patients being symptomatic but not bedridden, moderate as causing patients to be bedridden for ≥48 hours, and severe as requiring hospitalization. During screening, participants self-reported whether they were bedridden (and the length of time for) and/or whether they were hospitalized. Researchers then assigned the pre-defined severity classification based on responses. Recognizing that patients with existing health conditions are at risk of worse COVID-19 outcomes, an additional quota, wherein 20–30% of participants had a comorbid condition that pre-existed their COVID-19 diagnosis, was established to ensure the FLU-PRO Plus was appropriate for patients both with and without comorbidities.

### ***Procedure***

Ethics approval was granted by the New England Independent Review Board (#20203909). Individuals participated in one-to-one, 90-minute interviews via webcam or telephone, in a quiet and private location in their own home, at a time that was mutually convenient for the participant and interviewer. Interviews were conducted by trained and experienced qualitative researchers, using a semi-structured interview guide which included concept elicitation and cognitive debriefing techniques. All participants provided written informed consent prior to interview commencement.

Warm-up questions established rapport and gathered basic information about participants’ experiences with COVID-19. In the concept elicitation segment, participants were asked questions intended to generate descriptions of their symptoms, the impact of these symptoms, and their overall experience of COVID-19. The segment included a combination of pre-set, open-ended questions, and *ad hoc* probing for clarification of responses or further exploration of symptom experience.

In the cognitive debriefing segment, participants were instructed to complete the FLU-PRO Plus questionnaire while using a retrospective think-aloud method [4], where participants thought back to when they were ill, read aloud all elements of the instrument, and vocalized their decision‑making processes and answers to each FLU-PRO Plus item. Upon completion of the questionnaire, participants were asked structured questions to assess the relevance, comprehensibility, and comprehensiveness of the instrument. They were also asked to comment on the instructions, recall period, and response options of the FLU-PRO Plus, and to note any aspects they found confusing or inappropriate in measuring their symptoms.

### ***Analysis of qualitative data***

Interviews were audio-recorded and transcribed verbatim. Concept elicitation data were coded and analyzed using NVivo (v.12) software to identify patterns regarding relevant and important aspects of participants’ disease experience. In addition to assigning data to a set of *a priori* codes (i.e. codes developed in advance and linked to the interview guide), an adapted grounded theory approach was used, whereby additional codes were developed and refined as transcripts were analyzed [5]. The mix of coding methods ensured key aspects of COVID-19 symptoms were addressed while still leaving room for new descriptions and experiences to be considered.

Concept elicitation data were also used to map responses back to FLU-PRO Plus items. A forward and backward mapping approach confirmed that all symptoms related to COVID-19, as described by participants during interviews, were included and measured by FLU-PRO Plus items. A formal saturation analysis was undertaken for the concept elicitation portion of the interviews wherein coding of transcripts was evaluated for saturation in six sets of five transcripts. This analysis was conducted to confirm if sufficient interviews had been completed such that further interviews would not identify any new, relevant concepts.

Cognitive debriefing data were coded and analyzed using a Microsoft Excel workbook to systematically summarize participants’ feedback. More specifically, this workbook tracked the understandability, appropriateness, and relevance of items, response options, recall period, and instructions. All responses were evaluated by researchers and assigned codes to capture the type of feedback, and whether remarks were spontaneous or prompted after questioning by the interviewer. Comments pertaining to item comprehension and the ability to respond accurately to items were noted. Given the expected heterogeneity of the sample, all data were analyzed collectively and considered independently for each level of severity and for those with and without comorbidities.

**Supplementary Table 1** Responder definition

| **FLU-PRO Plus Item number** | **Responder definition** |
| --- | --- |
| 1, 2, 5, 10, 15, 19, 22, 24, 26 | Score of “Not at all” |
| 29, 23 | Score of no more than “Somewhat” |
| 31, 32 | Score of “0 times” |
| 33, 34 | Score of “Yes” |

A sustained response is defined as ≥48 hours

**Supplementary Table 2** Exemplary participants quotes - FLU-PRO Plus item mapping

| **FLU-PRO Symptom** | **Exemplary participant quotes** |
| --- | --- |
| Congested or stuffy nose | I was stuffy and couldn’t blow my nose. (Pt 4, Female, 56 y/o)  I was so stuffed up and… and couldn’t breathe and felt like congestion the whole time. (Pt 23, Female, 60 y/o) |
| Runny or dripping nose | My nose was very runny and drippy during most of the day, on most of the days that I was sick. (Pt 15, Female, 34 y/o)  During that time period, it ran a lot. I had that lovely, chapped nose type thing. And went through an awful lot of tissues. (Pt 22, Female, 60 y/o) |
| Sinus pressure | You felt like you were sucking up water through your nose. That’s how I, the, the only way I can explain it. (Pt 5, Female, 38 y/o)  It felt like somebody was squeezing my—my nose with a knot being in my nose. Um, that’s how I describe it, you know. (Pt 24, Male, 32 y/o) |
| Difficulty swallowing | I do remember having difficulty swallowing, like food. Not necessarily beverages. Although even tea, you know, it just, I would say, I would say, “Quite a bit.” I couldn’t really swallow. (Pt 4, Female, 56 y/o)  My throat swelled up and, it was very painful. As I said, I felt like I swallowed glass. And, almost immediately, that’s when I started the lemon water and honey to try and soothe it. (Pt 30, Female, 56 y/o) |
| Scratchy or itchy throat | You feel your tonsils back there, and you’re not supposed to feel them. You know, just, there, it’s something there that’s kind of rubbing against the, uh, back sides of your throat that shouldn’t be. And that’s, that’s how it felt. (Pt 21, Female, 70 y/o)  I felt like I had that constant tickle that would make you kind of like, you know, cough, or choke, or clear your throat. (Pt 22, Female, 60 y/o) |
| Sore or painful throat | Well, it was, it was very sore. I only wanted to drink stuff or, and if it got dry, then it was even worse, like if I fell asleep. (Pt 16, Female, 60 y/o)  woke up and it just felt like I had swallowed glass. My throat was just on fire. And I was just, you know, uh, just, I guess flu-like symptoms, if you will… You know, just starting to feel achy, and my throat was killing me. (Pt 30, Female, 56 y/o) |
| Eyes sensitive to light | I sit in my room, with all my lights off, for most of the time. Even in the hospital, they shut all the lights in my room. I was not exposed to light. Only if I went outside, uhm, or driving home from the hospital. (Pt 1, Male, 28 y/o)  I wanted it to be dark. I didn’t want like the curtains open, I didn’t want the lights on. I needed it to be dark. (Pt 29, Female, 57 y/o) |
| Sore or painful eyes | I do remember like kind of having that like heavy feeling on my eyes. My eyes feeling a little bit heavy during the day or just like puffy, that allergy kind of feeling that you get. (Pt 1, Male, 28 y/o)  My eyes hurt, they were irritated, felt like I had foreign bodies, but it wasn’t the kind of squinty thing that one would do… Gross and disgusting [laughs] and puffy… and, yes, that was not a specific disturbance. This was just…“I don’t like my eyeballs right now.” (Pt 26, Female, 66 y/o) |
| Teary or watery eyes | They were a little, a little runny. (Pt 19, Male, 62 y/o)  There was, just some tearing up and needed tissues just a little bit. (Pt 23, Female, 60 y/o) |
| Chest congestion | I felt very congested in my chest. Like, I actually felt it in my lungs, like I couldn’t breathe. (Pt 4, Female, 56 y/o)  You just feel like, the rattle of, like mucus in there somehow. I’ll say, somewhat towards the end of it when I was coughing up the phlegm. (Pt 14, Male, 30 y/o) |
| Chest tightness | It felt like there was a, you know, kind of a rubber band, you know I think I read it—people saying it’s like a rubber band, I’m like, “That’s a good analogy,” around my chest. (Pt 9, Male, 46 y/o)  I would feel like pressure, yeah. But I, I remember that I would make reference to, and I guess I, maybe I made reference to it as feeling pressure. (Pt 10, Male, 41 y/o) |
| Dry or hacking cough | It wasn’t, you know, it wasn’t a productive cough. Now I still get it with a little bit of tickle as I’m talking. (Pt 9, Male, 46 y/o)  It wasn’t the kind of cough like, like you feel like your chest is heavy, like with a chest cold. It wasn’t that. It was just like a dry cough. And not, it didn’t expel anything. It didn’t feel like I needed to expel anything. (Pt 29, Female, 57 y/o) |
| Trouble breathing | I could not deep breathe at all. Now, you know, almost a month later, I’m still having difficulty deep breathing. But at the time, I was, I could not breathe properly. I felt I could not catch my breath at all. (Pt 4, Female, 56 y/o)  I would realize that, at night sometimes, we’ll both wake up gasping for air….And we’ll realize that like, you know, we weren’t taking in any, any breaths. It, it was like kind of like we had, you know, sleep apnea or something. (Pt 14, Male, 30 y/o) |
| Wet or loose cough | I was coughing up the phlegm at that point. (Pt 14, Male, 30 y/o)  Coughing, um, to get rid of that stuff from down my throat so it wasn’t going to go into my lungs. (Pt 26, Female, 66 y/o |
| Felt nauseous | Like you don’t necessarily feel like, you’re going to throw up, like when you experience nausea. Like when I did experience that nausea, I didn’t necessarily, you know, I didn’t feel like there was a, you know, the threat of, of vomiting. (Pt 2, Female, 32 y/o)  I did have a couple of runs to the bathroom, thinking that was going to be possible. Most of the time I didn’t throw up because there was not very much in there to give back other than fluid. (Pt 26, Female, 66 y/o) |
| Stomach ache | My stomach started hurting… And it was just like when we on the mend, all of a sudden our stomachs started acting crazy. And it was just, uh, cramping and that kind of, that kind of feeling, not feeling very well. (Pt 5, Female, 38 y/o)  Just that kind of a stomach ache, you know, where you’re just feeling like that rumbling and you’re just like off and you’re just like… So, again, it’s, it’s a little bit different to me. I would, if I had to say something, I’d say quite a bit you know, intestinal distress. (Pt 30, Female, 56 y/o) |
| Felt dizzy | I remember waking up like during the night and kind of like having that very vertigo-ish [phonetic] feeling, like even just laying down. (Pt 30, Female, 56 y/o)  I would have to collect myself. I’d have to sit down, take deep breaths. I’d feel myself get pale. And, uh, just one time, I did have like a tunnel feeling because I didn’t know if I was going to pass out. (Pt 5, Female, 38 y/o) |
| Head congestion | As far as the, the, uh, head, head congestion, uh, you know, my head was very, uh, heavy, and I did feel the congestion in my head. (Pt 19, Male, 62 y/o)  It was, it, it hurt. I mean, it was a bad headache. It was almost like the kind where you want to rub it. It was bad. (Pt 16, Female, 60 y/o) |
| Headache | Like the headache was horrible. It was a headache I had never experienced, so I knew something wasn’t right. It was, it was brutal. It felt like your head was in a vice. (Pt 13, Male, 47 y/o)  But it’s just kind of a persistent in the back of my head that’s kind of annoying. Uh, headache is probably, just because it won’t go away for a couple of days..So, I would rank that relatively high. On an annoying scale of, you know, one to ten, I’d put that up there, uhm, as an eight. (Pt 9, Male, 46 y/o) |
| Body aches or pains | Definitely the body pains hurt quite the most. Uhm, because like if there was like any, any small movement, it felt like, you just had like crazy charley horses and, just being sore from the gym. But this was like, this was to another level. And like the back pain was, was hurting a lot. Like any small movement, it like, it just, it just hurt.(Pt 14, Male, 30 y/o)  Because definitely, like my body just ached, you know. Because I kind of felt like in the first couple days, I kind of felt like I fell off the roof or something like that, you know. My body was sore, achy, you know. (Pt 25, Male, 55 y/o) |
| Chills or shivering | I had the chills in the first couple of days. Uh, I was, uh, you know, not really a high fever but just the chills up and down my body. (Pt 5, Female, 38 y/o)  And the chills. The chill, the chill factor was probably the third most bothersome. Like I just couldn't stay warm…Yes, I was always cold. (Pt 11, Male, 70 y/o) |
| Felt cold | I felt very cold, so I used to have to keep warm. I used… Normally, I don’t wear socks when I go to sleep, but I used to wear my socks because I felt like I was pretty cold. (Pt 19, Male, 62 y/o)  There were definitely a couple of days where I felt like I couldn’t get warm no matter what I did. (Pt 15, Female, 34 y/o) |
| Felt hot | When the fever would come back, then I would start to feel hot. (Pt 3, Male, 45 y/o)  I did feel hot somewhat of the time, uhm, to the point that I would have to be on the opposite side of the room from the heating blanket because I just, I felt like I was burning up. (Pt 14, Male, 30 y/o) |
| Lack of appetite | I was able to eat but I, I, I couldn’t eat a full meal. I just, I just wasn’t hungry. (Pt 7, Male, 53 y/o)  If my husband didn’t feed me, I would not have eaten. (Pt 22, Female, 60 y/o) |
| Sleeping more than usual | It was like you would fall asleep and just lose time, that kind of sleep. Like usually I’ll dream or I wake up a couple of times, but it was not like that. It was I work up, it was sunny out, and I kind of like had to figure out where I was and that kind of, like reorient myself. (Pt 5, Female, 38 y/o)  I laid like a lump on a wall in bed for 16 hours a day for about a week. That was pretty much it. (Pt 11, Male, 70 y/o) |
| Sweating | There was some sweating at night and during the day when I slept. I would wake up sweating. (Pt 21, Female, 70 y/o)  I would sweat and literally wet the sheets, have to change everything, even though I couldn’t even get up, I was exhausted. (Pt 4, Female, 56 y/o) |
| Weak or tired | I live in a two-story. So me just going downstairs to grab something, it was pretty exhausting. Just because I didn’t have much energy to do much. (Pt 12, Female, 32 y/o)  I just didn’t feel like doing anything. And getting out of bed was a chore. It’s like you just lay there, you’re awake, but you just didn’t want to get up. (Pt 18, Male, 69 y/o) |
| Coughed up mucus or phlegm | I have continued to have a cough, and it is not always a dry cough. Uh, you know, I’ve also had some buildup of phlegm, uh, a couple of times. (Pt 17, Female, 53 y/o)  A little bit of a cough. Uh, it, just mainly just from the sinus, like sinus dripping down into your throat … just trying to cough out that mucus. (Pt 8, Male, 51 y/o) |
| Coughing | It was definitely apparent throughout the whole time. So, I definitely was coughing more. (Pt 6, Male, 22 y/o)  The cough was really the most worrisome of all of the symptoms. The cough lasted probably a good two weeks before it finally went away. Like it took, it didn't completely go away for a good two weeks. (Pt 11, Male, 70 y/o) |
| Sneezing | When I was getting, going into COVID, I sneezed a lot, uhm, now that I think of it, a lot more than normal, which is one thing I do when I’m starting to get something. (Pt 21, Female, 70 y/o)  It wasn’t consistent, it was just at certain times, I would just start sneezing and have several sneezes in a row. Blow my nose and then it might not do it again, until, you know, another day. (Pt 22, Female, 60 y/o) |
| Diarrhea | I had diarrhea. That came on pretty strong. I mean it was like water. And I ended up losing seven pounds in that couple weeks. (Pt 27, Female, 57 y/o)  Every day, multiple times a day for at least the first two weeks. And really, the first month, I still wasn’t right. Uhm, it was never anything where, it was never anything where I soiled myself or had to change. You know, like I still had control, uhm, but it was every day. (Pt 30, Female, 56 y/o) |
| Vomit | I threw up a few times. But I really, I didn’t want to eat, I didn’t even want to drink the tea. So, I guess nausea. It was more, I did throw up, maybe two times. Like I couldn’t really… but like I said, I wasn’t really eating either, so. (Pt 4, Female, 56 y/o)  Food was not going down well. Some coming back up…Um, it was how often I was running to the bathroom feeling like I could as opposed to, um, how many times did I actually bring something up. (Pt 26, Female, 66 y/o) |
| Loss of smell | I’m noticing that I’m not smelling things as strongly as I normally would. Like I had a candle burning the other day like right next to me, and I did not smell it at all and like would just get like kind of like whiffs of it. (Pt 15, Female, 34 y/o)  I have a face wash in the shower when I get up every morning, and it’s like a, it’s supposed to be morning burst or whatever. You know, it smells like oranges…..But, uhm, and I couldn’t smell it. So, then I like started running around the house trying to smell things. Like I couldn’t smell the coffee. (Pt 20, Female, 56 y/o) |
| Loss of taste | But only in the front of my tongue, like the anterior of my tongue, but I actually didn’t realize that. It took me like a day or so to even realize those things, uh, because it wasn’t a complete loss. (Pt 2, Female, 32 y/o)  The first thing I noticed when I, when I lost my, uh, sense of taste…And I’m putting all my spice and things in, and I’m like, “Wow, this is my best chili ever.” I said, “This is really good.” Put it in a bowl, I sat down and went to eat, I go, “What happened?” (Pt 18, Male, 69 y/o) |
| Brain fog | It’s kind of hard to explain. But yeah, uh, definitely just, uh just couldn’t really, just couldn’t really think straight, couldn’t really keep a, couldn’t really juggle more than one thought. I don’t know. It’s really hard to explain that kind of clouded kind of feeling. (Pt 1, Male, 28 y/o)  The fogginess just simply slows me down. So, in other words, you know, when you, like right now, what I did right now, and let’s just say it took me 10 minutes or 15 minutes to figure it out and answer my questions, coming out I still would have given you those same answers, but it would have taken me 30 minutes. (Pt 10, Male, 41 y/o) |

**Supplementary Table 3** Known-groups validity of FLU-PRO Plus scores by WPAI impairment on regular activities at Day 1

|  | **WPAI Impairment Regular Activities** | | | | | | | **Overall F-test** | | **p-value for pairwise comparisons^1,2^** |
| --- | --- | --- | --- | --- | --- | --- | --- | --- | --- | --- |
|  | **Total N** | **70–100 impairment** | | **40–60 impairment** | | **0–30 impairment** | |  |  |  |
|  |  | **N** | **LS mean (SE)** | **N** | **LS mean (SE)** | **N** | **LS mean (SE)** | **F** | **p-value** |  |
| **Nose** | 350 | 169 | 1.5 (0.1) | 91 | 1.2 (0.1) | 90 | 1.0 (0.1) | 11.05 | <0.0001 | 1 vs 2: 0.0535 1 vs 3: <0.0001 2 vs 3: 0.1569 |
| **Throat** | 350 | 169 | 1.3 (0.1) | 91 | 0.8 (0.1) | 90 | 0.7 (0.1) | 16.20 | <0.0001 | 1 vs 2: 0.0002 1 vs 3: <0.0001 2 vs 3: 0.7046 |
| **Eyes** | 350 | 169 | 1.1 (0.1) | 91 | 0.8 (0.1) | 90 | 0.4 (0.1) | 19.59 | <0.0001 | 1 vs 2: 0.0161 1 vs 3: <0.0001 2 vs 3: 0.0147 |
| **Chest/Respiratory** | 350 | 169 | 1.4 (0.1) | 91 | 1.2 (0.1) | 90 | 0.8 (0.1) | 19.93 | <0.0001 | 1 vs 2: 0.0971 1 vs 3: <0.0001 2 vs 3: 0.0015 |
| **Gastrointestinal** | 350 | 169 | 0.8 (0.1) | 91 | 0.7 (0.1) | 90 | 0.4 (0.1) | 12.39 | <0.0001 | 1 vs 2: 0.2161 1 vs 3: <0.0001 2 vs 3: 0.0188 |
| **Body/Systemic** | 350 | 169 | 1.7 (0.1) | 91 | 1.4 (0.1) | 90 | 0.9 (0.1) | 32.60 | <0.0001 | 1 vs 2: 0.0042 1 vs 3: <0.0001 2 vs 3: 0.0002 |
| **Taste/Smell** | 350 | 169 | 2.1 (0.1) | 91 | 1.9 (0.2) | 90 | 1.5 (0.2) | 3.53 | 0.0303 | 1 vs 2: 0.5927 1 vs 3: 0.0304 2 vs 3: 0.3575 |
| **FLU-PRO total score** | 350 | 169 | 1.4 (0.0) | 91 | 1.1 (0.1) | 90 | 0.7 (0.1) | 32.82 | <0.0001 | 1 vs 2: 0.0017 1 vs 3: <0.0001 2 vs 3: 0.0005 |
| **FLU-PRO Plus total score** | 350 | 169 | 1.5 (0.0) | 91 | 1.2 (0.1) | 90 | 0.8 (0.1) | 32.37 | <0.0001 | 1 vs 2: 0.0021 1 vs 3: <0.0001 2 vs 3: 0.0005 |

^1^Pairwise comparisons between LS means were performed using Scheffe’s test adjusting for multiple comparisons.

^2^1 refers to the 70–100 impairment column; 2 refers to the 40–60 impairment column; 3 refers to the 0–30 impairment column.

*LS* least squares *SE* standard error *WPAI* Work Productivity and Activity Impairment Questionnaire

**Supplementary Table 4** Known-groups validity of FLU-PRO Plus scores by WPAI impairment on regular activities at Day 15

|  | **WPAI Impairment Regular Activities** | | | | | | | **Overall F-test** | | **p-value for pairwise comparisons^1,2^** |
| --- | --- | --- | --- | --- | --- | --- | --- | --- | --- | --- |
|  | **Total N** | **70–100 impairment** | | **40–60 impairment** | | **0–30 impairment** | |  |  |  |
|  |  | **N** | **LS mean (SE)** | **N** | **LS mean (SE)** | **N** | **LS mean (SE)** | **F** | **p-value** |  |
| **Nose** | 231 | 57 | 0.5 (0.1) | 42 | 0.5 (0.1) | 132 | 0.2 (0.0) | 16.66 | <0.0001 | 1 vs 2: 0.8146 1 vs 3: <0.0001 2 vs 3: 0.0007 |
| **Throat** | 231 | 57 | 0.2 (0.1) | 42 | 0.3 (0.1) | 132 | 0.1 (0.0) | 5.74 | 0.0037 | 1 vs 2: 0.5360 1 vs 3: 0.1187 2 vs 3: 0.0080 |
| **Eyes** | 231 | 57 | 0.3 (0.1) | 42 | 0.2 (0.1) | 132 | 0.1 (0.0) | 8.67 | 0.0002 | 1 vs 2: 0.6460 1 vs 3: 0.0006 2 vs 3: 0.0547 |
| **Chest/Respiratory** | 231 | 57 | 0.7 (0.1) | 42 | 0.6 (0.1) | 132 | 0.2 (0.0) | 26.69 | <0.0001 | 1 vs 2: 0.3455 1 vs 3: <0.0001 2 vs 3: <0.0001 |
| **Gastrointestinal** | 231 | 57 | 0.1 (0.0) | 42 | 0.2 (0.0) | 132 | 0.1 (0.0) | 2.84 | 0.0603 | 1 vs 2: 0.7186 1 vs 3: 0.3573 2 vs 3: 0.0873 |
| **Body/Systemic** | 231 | 57 | 0.6 (0.0) | 42 | 0.4 (0.1) | 132 | 0.1 (0.0) | 32.54 | <0.0001 | 1 vs 2: 0.2285 1 vs 3: <0.0001 2 vs 3: <0.0001 |
| **Taste/Smell** | 231 | 57 | 1.3 (0.2) | 42 | 0.6 (0.2) | 132 | 0.6 (0.1) | 4.42 | 0.0131 | 1 vs 2: 0.1081 1 vs 3: 0.0160 2 vs 3: 0.9867 |
| **FLU-PRO total score** | 231 | 57 | 0.5 (0.0) | 42 | 0.4 (0.0) | 132 | 0.1 (0.0) | 29.59 | <0.0001 | 1 vs 2: 0.5120 1 vs 3: <0.0001 2 vs 3: <0.0001 |
| **FLU-PRO Plus total score** | 231 | 57 | 0.5 (0.0) | 42 | 0.4 (0.1) | 132 | 0.2 (0.0) | 26.75 | <0.0001 | 1 vs 2: 0.3020 1 vs 3: <0.0001 2 vs 3: 0.0001 |

^1^Pairwise comparisons between LS means were performed using Scheffe’s test adjusting for multiple comparisons.

^2^1 refers to the 70–100 impairment column; 2 refers to the 40–60 impairment column; 3 refers to the 0–30 impairment column.

*LS* least squares *SE* standard error *WPAI* Work Productivity and Activity Impairment Questionnaire

**Supplementary Table 5** Known-groups validity of FLU-PRO Plus scores by WPAI impairment on regular activities at Day 29

|  | **WPAI Impairment Regular Activities** | | | | | | | **Overall F-test** | | **p-value for pairwise comparisons^1,2^** |
| --- | --- | --- | --- | --- | --- | --- | --- | --- | --- | --- |
|  | **Total N** | **70–100 impairment** | | **40–60 impairment** | | **0–30 impairment** | |  |  |  |
|  |  | **N** | **LS mean (SE)** | **N** | **LS mean (SE)** | **N** | **LS mean (SE)** | **F** | **p-value** |  |
| **Nose** | 291 | 30 | 0.6 (0.1) | 46 | 0.3 (0.1) | 215 | 0.1 (0.0) | 18.54 | <0.0001 | 1 vs 2: 0.0020 1 v. 3: <0.0001 2 v. 3: 0.1327 |
| **Throat** | 291 | 30 | 0.3 (0.1) | 46 | 0.1 (0.0) | 215 | 0.0 (0.0) | 5.80 | 0.0034 | 1 vs 2: 0.3807 1 vs 3: 0.0078 2 vs 3: 0.2139 |
| **Eyes** | 291 | 30 | 0.1 (0.0) | 46 | 0.2 (0.0) | 215 | 0.0 (0.0) | 6.33 | 0.0020 | 1 vs 2: 0.9320 1 vs 3: 0.1011 2 vs 3: 0.0083 |
| **Chest/Respiratory** | 291 | 30 | 0.5 (0.1) | 46 | 0.4 (0.1) | 215 | 0.1 (0.0) | 14.53 | <0.0001 | 1 vs 2: 0.1820 1 vs 3: <0.0001 2 vs 3: 0.0088 |
| **Gastrointestinal** | 291 | 30 | 0.2 (0.0) | 46 | 0.1 (0.0) | 215 | 0.0 (0.0) | 7.45 | 0.0007 | 1 vs 2: 0.5116 1 vs 3: 0.0039 2 vs 3: 0.0629 |
| **Body/Systemic** | 291 | 30 | 0.6 (0.1) | 46 | 0.2 (0.0) | 215 | 0.1 (0.0) | 31.31 | <0.0001 | 1 vs 2: <0.0001 1 vs 3: <0.0001 2 vs 3: 0.0388 |
| **Taste/Smell** | 291 | 30 | 0.6 (0.2) | 46 | 0.3 (0.2) | 215 | 0.4 (0.1) | 0.54 | 0.5861 | 1 vs 2: 0.6433 1 vs 3: 0.6184 2 vs 3: 0.9838 |
| **FLU-PRO total score** | 291 | 30 | 0.5 (0.0) | 46 | 0.2 (0.0) | 215 | 0.1 (0.0) | 25.15 | <0.0001 | 1 vs 2: 0.0033 1 vs 3: <0.0001 2 vs 3: 0.0073 |
| **FLU-PRO Plus total score** | 291 | 30 | 0.5 (0.1) | 46 | 0.2 (0.0) | 215 | 0.1 (0.0) | 21.97 | <0.0001 | 1 vs 2: 0.0045 1 vs 3: <0.0001 2 vs 3: 0.0196 |

^1^Pairwise comparisons between LS means were performed using Scheffe’s test adjusting for multiple comparisons.

^2^1 refers to the 70–100 impairment column; 2 refers to the 40–60 impairment column; 3 refers to the 0–30 impairment column.

*LS* least squares *SE* standard error *WPAI* Work Productivity and Activity Impairment Questionnaire

**Supplementary Table 6** Known-groups validity of FLU-PRO Plus scores by WPAI impairment on regular activities at Week 8

|  | **WPAI Impairment Regular Activities** | | | | | | | **Overall F-test** | | **p-value for pairwise comparisons^1,2^** |
| --- | --- | --- | --- | --- | --- | --- | --- | --- | --- | --- |
|  | **Total N** | **70–100 impairment** | | **40–60 impairment** | | **0–30 impairment** | |  |  |  |
|  |  | **N** | **LS mean (SE)** | **N** | **LS mean (SE)** | **N** | **LS mean (SE)** | **F** | **p-value** |  |
| **Nose** | 358 | 36 | 0.5 (0.1) | 44 | 0.4 (0.1) | 278 | 0.2 (0.0) | 13.97 | <0.0001 | 1 vs 2: 0.3416 1 vs 3: <0.0001 2 vs 3: 0.0099 |
| **Throat** | 358 | 36 | 0.3 (0.0) | 44 | 0.2 (0.0) | 278 | 0.0 (0.0) | 15.44 | <0.0001 | 1 vs 2: 0.0472 1 vs 3: <0.0001 2 vs 3: 0.0660 |
| **Eyes** | 358 | 36 | 0.4 (0.1) | 44 | 0.2 (0.0) | 278 | 0.1 (0.0) | 22.47 | <0.0001 | 1 vs 2: 0.0006 1 vs 3: <0.0001 2 vs 3: 0.1895 |
| **Chest/Respiratory** | 358 | 36 | 0.4 (0.1) | 44 | 0.2 (0.1) | 278 | 0.1 (0.0) | 18.56 | <0.0001 | 1 vs 2: 0.0296 1 vs 3: <0.0001 2 vs 3: 0.0333 |
| **Gastrointestinal** | 358 | 36 | 0.2 (0.0) | 44 | 0.1 (0.0) | 278 | 0.0 (0.0) | 24.30 | <0.0001 | 1 vs 2: 0.0219 1 vs 3: <0.0001 2 vs 3: 0.0052 |
| **Body/Systemic** | 358 | 36 | 0.5 (0.0) | 44 | 0.3 (0.0) | 278 | 0.1 (0.0) | 62.84 | <0.0001 | 1 vs 2: 0.0025 1 vs 3: <0.0001 2 vs 3: <0.0001 |
| **Taste/Smell** | 358 | 36 | 0.9 (0.2) | 44 | 0.4 (0.1) | 278 | 0.2 (0.1) | 7.46 | 0.0007 | 1 vs 2: 0.0590 1 vs 3: 0.0007 2 vs 3: 0.6758 |
| **FLU-PRO total score** | 358 | 36 | 0.4 (0.0) | 44 | 0.3 (0.0) | 278 | 0.1 (0.0) | 45.70 | <0.0001 | 1 vs 2: 0.0018 1 vs 3: <0.0001 2 vs 3: <0.0001 |
| **FLU-PRO Plus total score** | 358 | 36 | 0.5 (0.0) | 44 | 0.3 (0.0) | 278 | 0.1 (0.0) | 44.60 | <0.0001 | 1 vs 2: 0.0009 1 vs 3: <0.0001 2 vs 3: <0.0001 |

^1^Pairwise comparisons between LS means were performed using Scheffe’s test adjusting for multiple comparisons.

^2^1 refers to the 70–100 impairment column; 2 refers to the 40–60 impairment column; 3 refers to the 0–30 impairment column.

*LS* least squares *SE* standard error *WPAI* Work Productivity and Activity Impairment Questionnaire

**Supplementary Table 7** Known-groups validity of FLU-PRO Plus scores by WPAI impairment on regular activities at Week 12

|  | **WPAI Impairment Regular Activities** | | | | | | | **Overall F-test** | | **p-value for pairwise comparisons^1,2^** |
| --- | --- | --- | --- | --- | --- | --- | --- | --- | --- | --- |
|  | **Total N** | **70–100 impairment** | | **40–60 impairment** | | **0–30 impairment** | |  |  |  |
|  |  | **N** | **LS mean (SE)** | **N** | **LS mean (SE)** | **N** | **LS mean (SE)** | **F** | **p-value** |  |
| **Nose** | 304 | 34 | 0.6 (0.1) | 26 | 0.4 (0.1) | 244 | 0.2 (0.0) | 14.54 | <0.0001 | 1 vs 2: 0.1431 1 vs 3: <0.0001 2 vs 3: 0.1147 |
| **Throat** | 304 | 34 | 0.2 (0.1) | 26 | 0.1 (0.1) | 244 | 0.1 (0.0) | 4.14 | 0.0169 | 1 vs 2: 0.2490 1 vs 3: 0.0170 2 vs 3: 0.9079 |
| **Eyes** | 304 | 34 | 0.3 (0.1) | 26 | 0.1 (0.1) | 244 | 0.1 (0.0) | 4.03 | 0.0188 | 1 vs 2: 0.2330 1 vs 3: 0.0188 2 vs 3: 0.9384 |
| **Chest/Respiratory** | 304 | 34 | 0.3 (0.1) | 26 | 0.3 (0.1) | 244 | 0.1 (0.0) | 11.64 | <0.0001 | 1 vs 2: 0.9856 1 vs 3: 0.0013 2 vs 3: 0.0026 |
| **Gastrointestinal** | 304 | 34 | 0.2 (0.1) | 26 | 0.2 (0.1) | 244 | 0.0 (0.0) | 7.50 | 0.0007 | 1 vs 2: 0.7783 1 vs 3: 0.0031 2 vs 3: 0.1001 |
| **Body/Systemic** | 304 | 34 | 0.5 (0.0) | 26 | 0.3 (0.1) | 244 | 0.1 (0.0) | 36.57 | <0.0001 | 1 vs 2: 0.0120 1 vs 3: <0.0001 2 vs 3: 0.0029 |
| **Taste/Smell** | 304 | 34 | 0.3 (0.2) | 26 | 0.5 (0.2) | 244 | 0.2 (0.1) | 0.76 | 0.4706 | 1 vs 2: 0.7894 1 vs 3: 0.9311 2 vs 3: 0.4853 |
| **FLU-PRO total score** | 304 | 34 | 0.4 (0.0) | 26 | 0.3 (0.0) | 244 | 0.1 (0.0) | 25.43 | <0.0001 | 1 vs 2: 0.0962 1 vs 3: <0.0001 2 vs 3: 0.0067 |
| **FLU-PRO Plus total score** | 304 | 34 | 0.4 (0.0) | 26 | 0.3 (0.0) | 244 | 0.1 (0.0) | 22.98 | <0.0001 | 1 vs 2: 0.1778 1 vs 3: <0.0001 2 vs 3: 0.0061 |

^1^Pairwise comparisons between LS means were performed using Scheffe’s test adjusting for multiple comparisons.

^2^1 refers to the 70–100 impairment column; 2 refers to the 40–60 impairment column; 3 refers to the 0–30 impairment column.

*LS* least squares *SE* standard error *WPAI* Work Productivity and Activity Impairment Questionnaire

**Supplementary Table 8** Responsiveness of FLU-PRO Plus scores by change in WPAI impairment on regular activities from Day 1 to Day 29

|  | **WPAI Impairment** | | | | | **Overall F-test** | | **Effect size** |
| --- | --- | --- | --- | --- | --- | --- | --- | --- |
|  | **Total N** | **≥-20% worsened/stable** | | **<-20% improved** | |  |  |  |
|  |  | **N** | **LS mean (SE)** | **N** | **LS mean (SE)** | **F** | **p-value** |  |
| **Nose** | 173 | 82 | -0.9 (0.1) | 91 | -1.2 (0.1) | 4.26 | 0.0406 | -0.31 |
| **Throat** | 173 | 82 | -0.6 (0.1) | 91 | -1.1 (0.1) | 11.97 | 0.0007 | -0.53 |
| **Eyes** | 173 | 82 | -0.4 (0.1) | 91 | -1.0 (0.1) | 18.21 | <0.0001 | -0.65 |
| **Chest/Respiratory** | 173 | 82 | -0.8 (0.1) | 91 | -1.2 (0.1) | 11.18 | 0.0010 | -0.51 |
| **Gastrointestinal** | 173 | 82 | -0.4 (0.1) | 91 | -0.8 (0.1) | 15.48 | 0.0001 | -0.60 |
| **Body/Systemic** | 173 | 82 | -0.8 (0.1) | 91 | -1.5 (0.1) | 45.70 | <0.0001 | -1.03 |
| **Taste/Smell** | 173 | 82 | -1.2 (0.2) | 91 | -2.0 (0.2) | 5.07 | 0.0257 | -0.34 |
| **FLU-PRO total score** | 173 | 82 | -0.7 (0.1) | 91 | -1.2 (0.1) | 32.54 | <0.0001 | -0.87 |
| **FLU-PRO Plus total score** | 173 | 82 | -0.7 (0.1) | 91 | -1.3 (0.1) | 32.54 | <0.0001 | -0.87 |

*LS* least squares *SE* standard error *WPAI* Work Productivity and Activity Impairment Questionnaire

**Supplementary Table 9** Responder definition with key outcomes from Day 1 to Day 15

|  | **Total N** | **FLU-PRO Plus responder** | | | | **Overall F-test** | |
| --- | --- | --- | --- | --- | --- | --- | --- |
|  |  | **No** | | **Yes** | |  |  |
|  |  | **N** | **Mean (SD)** | **N** | **Mean (SD)** | **F** | **p-value** |
| **SF-12 MCS** | 400 | 253 | 48.0 (10.1) | 147 | 55.2 (8.2) | 54.29 | <0.0001 |
| **SF-12 PCS** | 400 | 253 | 47.0 (8.7) | 147 | 54.0 (5.0) | 80.72 | <0.0001 |
| **SF-12 Role Physical domain** | 400 | 253 | 62.5 (27.1) | 147 | 89.4 (17.9) | 115.52 | <0.0001 |
| **SF-12 Vitality domain** | 400 | 253 | 46.2 (30.3) | 147 | 73.6 (31.2) | 74.32 | <0.0001 |
| **SF-12 General Health question** | 400 | 253 | 63.3 (22.0) | 147 | 75.7 (16.6) | 34.95 | <0.0001 |
| **Pre-COVID Health Supplemental question** | 399 | 253 | 3.4 (1.0) | 146 | 2.6 (0.9) | 69.29 | <0.0001 |
| **WPAI Overall work impairment (%)** | 81 | 55 | 54.1 (34.4) | 26 | 28.9 (23.2) | 11.49 | 0.0011 |
| **WPAI Work time missed (%)** | 164 | 119 | 51.0 (41.1) | 45 | 29.5 (38.4) | 9.31 | 0.0027 |
| **WPAI Activity impairment (%)** | 231 | 138 | 48.5 (30.5) | 93 | 23.0 (23.5) | 46.22 | <0.0001 |

*MCS* mental component summary score *PCS* physical component summary score *SD* standard deviation *SF-12* 12-item Short Form *WPAI* Work Productivity and Activity Impairment Questionnaire

**Supplementary Table 10** Responder definition with key outcomes from Day 1 to Day 29

|  | **Total N** | **FLU-PRO Plus responder** | | | | **Overall F-test** | |
| --- | --- | --- | --- | --- | --- | --- | --- |
|  |  | **No** | | **Yes** | |  |  |
|  |  | **N** | **Mean (SD)** | **N** | **Mean (SD)** | **F** | **p-value** |
| **SF-12 MCS** | 431 | 153 | 48.5 (10.3) | 278 | 55.5 (7.6) | 64.71 | <0.0001 |
| **SF-12 PCS** | 431 | 153 | 47.2 (9.0) | 278 | 53.4 (5.4) | 79.07 | <0.0001 |
| **SF-12 Role Physical domain** | 431 | 153 | 65.4 (28.9) | 278 | 86.6 (18.6) | 85.64 | <0.0001 |
| **SF-12 Vitality domain** | 431 | 153 | 46.4 (27.7) | 278 | 73.8 (29.6) | 88.58 | <0.0001 |
| **SF-12 General Health question** | 431 | 153 | 62.6 (22.8) | 278 | 73.8 (17.2) | 32.79 | <0.0001 |
| **Pre-COVID Health Supplemental question** | 428 | 153 | 3.4 (0.9) | 275 | 2.8 (0.8) | 50.75 | <0.0001 |
| **WPAI Overall work impairment (%)** | 109 | 42 | 36.3 (24.7) | 67 | 26.2 (24.5) | 4.35 | 0.0394 |
| **WPAI Work time missed (%)** | 163 | 66 | 20.4 (35.1) | 97 | 7.2 (19.6) | 9.38 | 0.0026 |
| **WPAI Activity impairment (%)** | 291 | 98 | 36.8 (27.9) | 193 | 21.1 (21.0) | 28.84 | <0.0001 |

*MCS* mental component summary score *PCS* physical component summary score *SD* standard deviation *SF-12* 12-item Short Form *WPAI* Work Productivity and Activity Impairment Questionnaire

**Supplementary Table 11** Responder definition with key outcomes from Day 1 to Week 8

|  | **Total N** | **FLU-PRO Plus responder** | | | | **Overall F-test** | |
| --- | --- | --- | --- | --- | --- | --- | --- |
|  |  | **No** | | **Yes** | |  |  |
|  |  | **N** | **Mean (SD)** | **N** | **Mean (SD)** | **F** | **p-value** |
| **SF-12 MCS** | 391 | 143 | 48.1 (10.6) | 248 | 55.5 (7.8) | 61.32 | <0.0001 |
| **SF-12 PCS** | 391 | 143 | 47.8 (9.2) | 248 | 54.2 (5.8) | 71.44 | <0.0001 |
| **SF-12 Role Physical domain** | 391 | 143 | 68.0 (25.0) | 248 | 89.7 (18.5) | 95.94 | <0.0001 |
| **SF-12 Vitality domain** | 391 | 143 | 47.6 (27.1) | 248 | 73.6 (30.2) | 72.50 | <0.0001 |
| **SF-12 General Health question** | 391 | 143 | 62.3 (23.1) | 248 | 77.4 (18.6) | 49.39 | <0.0001 |
| **Pre-COVID Health Supplemental question** | 391 | 143 | 3.3 (0.8) | 248 | 2.6 (0.9) | 64.48 | <0.0001 |
| **WPAI Overall work impairment (%)** | 172 | 68 | 34.9 (29.4) | 104 | 15.5 (16.7) | 30.50 | <0.0001 |
| **WPAI Work time missed (%)** | 183 | 71 | 10.3 (22.9) | 112 | 3.8 (16.8) | 4.88 | 0.0284 |
| **WPAI Activity impairment (%)** | 358 | 126 | 37.1 (28.1) | 232 | 16.0 (17.7) | 75.40 | <0.0001 |

*MCS* mental component summary score *PCS* physical component summary score *SD* standard deviation *SF-12* 12-item Short Form *WPAI* Work Productivity and Activity Impairment Questionnaire

**Supplementary Table 12** Responder definition with key outcomes from Day 1 to Week 12

|  | **Total N** | **FLU-PRO Plus responder** | | | | **Overall F-test** | |
| --- | --- | --- | --- | --- | --- | --- | --- |
|  |  | **No** | | **Yes** | |  |  |
|  |  | **N** | **Mean (SD)** | **N** | **Mean (SD)** | **F** | **p-value** |
| **SF-12 MCS** | 319 | 123 | 48.2 (11.3) | 196 | 54.0 (8.8) | 26.27 | <0.0001 |
| **SF-12 PCS** | 319 | 123 | 47.9 (8.9) | 196 | 54.6 (5.7) | 65.30 | <0.0001 |
| **SF-12 Role Physical domain** | 319 | 123 | 67.8 (27.8) | 196 | 88.1 (20.1) | 57.22 | <0.0001 |
| **SF-12 Vitality domain** | 319 | 123 | 47.2 (28.3) | 196 | 70.8 (31.4) | 46.17 | <0.0001 |
| **SF-12 General Health question** | 319 | 123 | 59.8 (21.7) | 196 | 77.2 (18.7) | 57.79 | <0.0001 |
| **Pre-COVID Health Supplemental question** | 319 | 123 | 3.3 (1.0) | 196 | 2.5 (1.0) | 49.82 | <0.0001 |
| **WPAI Overall work impairment (%)** | 142 | 64 | 29.5 (25.5) | 78 | 16.0 (15.2) | 15.16 | 0.0002 |
| **WPAI Work time missed (%)** | 146 | 67 | 5.3 (15.0) | 79 | 5.1 (16.9) | 0.00 | 0.9497 |
| **WPAI Activity impairment (%)** | 304 | 117 | 36.0 (27.4) | 187 | 17.4 (17.0) | 53.28 | <0.0001 |

*MCS* mental component summary score *PCS* physical component summary score *SD* standard deviation *SF-12* 12-item Short Form *WPAI* Work Productivity and Activity Impairment Questionnaire

# References

1. Turner-Bowker D. M., Lamoureux R. E., Stokes J., Litcher-Kelly L., Galipeau N., Yaworsky A., Solomon J., Shields A. L. (2018) Informing a priori Sample Size Estimation in Qualitative Concept Elicitation Interview Studies for Clinical Outcome Assessment Instrument Development. *Value Health, 21*(7), 839-842. <https://doi.org/10.1016/j.jval.2017.11.014>.

2. Patrick D. L., Burke L. B., Gwaltney C. J., Leidy N. K., Martin M. L., Molsen E., Ring L. (2011). Content validity--establishing and reporting the evidence in newly developed patient-reported outcomes (PRO) instruments for medical product evaluation: ISPOR PRO good research practices task force report: part 1--eliciting concepts for a new PRO instrument. *Value in Health*, *14*(8), 967-977. https://doi.org/10.1016/j.jval.2011.06.014.

3. Patrick D. L., Burke L. B., Gwaltney C. J., Leidy N. K., Martin M. L., Molsen E., Ring L. (2011). Content validity--establishing and reporting the evidence in newly developed patient-reported outcomes (PRO) instruments for medical product evaluation: ISPOR PRO good research practices task force report: part 2--assessing respondent understanding. *Value in Health, 14*(8), 978-988. <https://doi.org/10.1016/j.jval.2011.06.013>.

4. Willis G. B. (2005). *Cognitive interviewing: a tool for improving questionnaire design*. University of California: SAGE Publications.

5. Vollstedt M., Rezat S. (2019). An introduction to grounded theory with a special focus on axial coding and the coding paradigm. In: G. Kaiser, N. Presmeg (Eds.), *Compendium for early career researchers in mathematics education*. (1st ed., pp. 81-100). Springer.
